# Evaluation of Public Involvement in Doctoral Research Using a Four‐Dimensional Theoretical Framework

**DOI:** 10.1111/hex.14149

**Published:** 2024-07-17

**Authors:** Piotr Teodorowski, Naheed Tahir, Saiqa Ahmed

**Affiliations:** ^1^ Faculty of Health Sciences and Sport University of Stirling Stirling UK; ^2^ ARC NWC Public Advisor

**Keywords:** doctoral research, PPI, public involvement, qualitative, review

## Abstract

**Background:**

Working together and co‐production with public advisors have become popular among health researchers. This practice extends to doctoral researchers who involve public advisors at different stages of their research or throughout their doctoral journey.

**Objective:**

A doctoral researcher and two public advisors jointly evaluated public involvement in doctoral research.

**Methods:**

Using the established public involvement evaluation framework by Gibson and colleagues, public advisors and a doctoral researcher mapped and evaluated their experiences of public involvement in doctoral research. The four‐dimensional framework allowed the authors to reflect on (1) the strength of the public voice, (2) the number of ways in which public advisors had an opportunity to get involved, (3) whether the discussion was about the public or organisation's (doctoral researcher, university or funder) concerns and (4) if the organisation changed or resisted feedback. Results are presented in a diagrammatic and narrative way.

**Results:**

Public advisors saw themselves as having a stronger voice in doctoral research than the doctoral researcher perceived. All agreed that there existed multiple ways for public advisors to be involved. Public advisors' feedback was taken on board, but it was also limited due to restrictions of what the doctoral programme allowed.

**Conclusion:**

Public advisors ensured that the doctoral research was more relevant to the public. The ongoing involvement also shaped the doctoral researcher's thinking and views.

**Patient and Public Involvement:**

Two public advisors were involved throughout the 3 years of this doctoral research. They co‐evaluated this involvement and are co‐authors of this paper.

## 1 Introduction

Public involvement has been embedded in health research and services [1, 2, 3, 4]. Many doctoral researchers have involved public advisors throughout their research, and several papers provide reflections and discussions on this practice [5, 6, 7, 8, 9, 10]. These papers come from a range of research fields and doctoral projects that utilised public involvement. They explored the experiences of service users with cerebral palsy [5], self‐management and patient activation [6], views of South Asian communities in their involvement in research [8] and self‐harm in older adults [10]. Papers used a case study approach [6, 9] and were reflection pieces [8, 10]. These were based on various sources such as notes, meeting minutes, correspondence and discussions with public advisors. Only one of these papers discussed public involvement during the Covid‐19 pandemic [5].

This paper contributes to the literature by evaluating public involvement in doctoral research during the COVID‐19 pandemic. Two public advisors and the doctoral researcher conducted the evaluation collaboratively, utilising Gibson and colleagues' four‐dimensional framework [11, 12]. To the best of the authors' knowledge, this is the first instance in which the four‐dimensional framework has been utilised to evaluate public involvement in doctoral research.

## 2 Background of Doctoral Thesis in the Context of Public Involvement

Technological advancements have enabled the development of big data research with the aim of improving healthcare and health inequalities [13, 14]. For example, the Liverpool City Region Civic Data Cooperative allows for the linkage of various data sets for research, including patient records and synthetic data, providing researchers with access to data for new projects [15]. However, there has been limited understanding of how to involve and engage the public around it. The present doctoral research explored how to involve and engage the public, especially seldom‐heard communities, in big data research. The literature was scoped by conducting a two‐stage review to understand what is known about this topic. Thereafter, three primary studies were conducted. The first project explored how researchers involved and engaged members of the public in big data research [16]. The second study looked at the perspective of seldom‐heard communities and interviewed Polish and South Asian communities in the United Kingdom, not previously involved as public contributors, to understand what could be put in place to get them involved in research [17]. The third project examined the #DataSavesLives campaign on Twitter/X and how the hashtag was used to engage with the public [18].

Before beginning doctoral research, P.T. worked for a charity, where he was involved in public and patient engagement sessions, thereby gaining an understanding of public involvement. This understanding was further developed through training sessions and webinars attended throughout the studies. The supervisors had prior experience involving public advisors in research. The doctoral researcher received further assistance from the public involvement manager with NIHR Applied Research Collaboration North West Coast (NIHR ARC NWC), who was a conduit between public advisors and researchers. No ethical approval for public involvement was required.

Three months into the PhD journey, the doctoral researcher recruited two public advisors through the Public Adviser Forum at NIHR ARC NWC. Since the PhD research did not require a specific lived experience as a patient of a particular health service, it was an appropriate place to find public advisors. Public Adviser Forum offered inductions (on being a public advisor), regular support and networking opportunities. Public advisors attended the Public Adviser Forum, regular local conferences, joint doctoral researchers and public advisors' journal club and were invited to participate in relevant training opportunities. Most of these activities were reimbursed, and any additional expenses were covered (e.g., travel costs for face‐to‐face meetings and document printing for online meetings if a public advisor preferred hard paper copies). There was an established process for reimbursing their time, supported by the administrator. This well‐established network of support ensured that there was no need for the doctoral researcher to provide any further training or explanation of what being a public advisor entailed, but rather focused on their involvement in the doctoral research.

The doctoral researcher initiated the involvement process, but it was a joint decision with public advisors on how much they were involved in the different research projects. The reason for letting public advisors choose their level of involvement was based on the premise that they might have restricted capacity or preference. Their personal circumstances might have only allowed a certain level of involvement, or public advisors could be interested in learning more about the specific methods or topics [19]. After discussing different options, public advisors were involved in the scoping and systematic (two‐stage) review, interviews with seldom‐heard communities and interviews with researchers. However, due to funding restraints, they were not able to participate in the Twitter project.

This paper presents the involvement process throughout the doctoral journey from the perspectives of the doctoral researcher and two public advisors. Their involvement in the review will now be discussed, followed by the qualitative studies. However, it is important to note that these activities often occurred simultaneously. Table 1, guided by the GRIPP2 reporting form [20], summarises the impact of involving public advisors in these research projects.

**Table 1:** Summary of the public involvement, training offered and impact in the doctoral research.

Project	Research activity	Public advisors' involvement	Training offered	Impact of involvement
Review	Design	Involved in designing review questions. Co‐designing the system logic model underpinning the review. Reviewing and commenting on review protocol. Co‐authorship of review protocol.	Training around big data research and reviews. Additional reading on reviews was offered for one public advisor.	Clarified the focus of the review and inclusion criteria through the development of a logic model. Additional search terms.
	Screening stage	Involved in jointly screening a sample of titles, abstracts and full papers.	Training before each screening stage: title, abstract and full paper.	Ensured appropriate inclusion of papers in relation to the review aims.
	Data extraction	Piloting and improving the data extraction form. Involvement in checking the extracted papers.	Training on data extraction and jointly extracting data from a sample of papers.	
	Analysis	Feedback on results.	None	Ensuring relevance to members of the public and how results relate to their experience as public advisors.
	Dissemination	Co‐authorship of the conference abstract.	None	Ensured public views in the output.
Qualitative studies	Design and ethics application	Involved in the ethics application. Reviewing participant information sheet, consent form and interview guide. Co‐designing the study ads. Co‐design and co‐chair public involvement sessions with the general public to pilot interview questions. Recruited participants for the South Asian involvement group.	None	Shaping the study aims. Ensuring lay language (no jargon) in all study materials. Creating appealing study ads to reach potential participants. Changes to the interview guide.
	Recruitment	Acted as gatekeepers to assist with the recruitment of South Asian interviewees.	None	Assisted with reaching South Asian participants. Recruitment of participants for each group (Polish and South Asian participants' recruitment targets were met).
	Analysis	Coding of one interview per participant group. Involved in shaping themes (e.g., through joint meetings with the supervisory team).	Training around the reflexive thematic analysis and being reflective.	Involvement shaped the results and the priorities for the discussion section.
	Dissemination	Reviewing and commenting on papers. Co‐authorship of papers. Co‐authorship of conference abstract and co‐presenting it. Advised on disseminating study results to the public.	Additional reading on research methods was identified for one public advisor.	Assisted in identifying the target audience for dissemination. Ensured public views in all outputs.

### 2.1 Scoping and Systematic Reviews

The review consisted of two stages. A scoping review of public involvement and engagement in big data research was conducted at Stage 1. In Stage 2, a systematic review was completed out of the identified papers, examining the delivery and effectiveness of strategies for involvement and engagement. This was the first of the three PhD projects that the public advisors became involved in. Co‐authored by public advisors, the review protocol [21] and the conference abstract have been published elsewhere [22]. As public advisors might require support and training to be fully involved in research [23, 24], and big data research can be a complex topic for the general public [25], during the first meeting, the doctoral researcher designed and delivered training on what big data are to ensure their familiarity with it. This consisted of explaining what big data are using everyday language and outlining the issues and challenges regarding big data research. This made the public advisors feel confident in discussing this topic. Thereafter, the doctoral researcher built the public involvement and engagement system logic model in big data research used to underpin the review. Feedback was collected from the public advisors during that first meeting to develop the initial logic model. The draft model was shared and refined with everyone involved. Sections identified through public advisors' input were marked on the model to recognise their contributions.

The doctoral researcher discussed with public advisors if they would be interested in getting involved in both stages of the review. They responded positively and became co‐authors of the review protocol. As co‐authors, the public advisors offered more practical implications for members of the public.

It was agreed that public advisors would contribute at every review stage. Before the title, abstract and full paper screening, the doctoral researcher conducted additional training sessions, focusing on both theoretical and practical research skills. For example, the doctoral researcher explained the process during the title screening stage. Subsequently, 100 titles were collectively screened as a group, and discussions were held regarding their inclusion in the abstract stage. Afterwards, the doctoral researcher sent everyone their titles for screening within their own time. Each person completed their screening, and the doctoral researcher compared the results to identify disagreements. The group reconvened to discuss these disagreements before moving on to the next stage. This process was repeated for the abstract and full paper stages. Most disagreements occurred during the title and abstract stages, as it was often unclear whether the paper included any aspect of public involvement based on the provided information. The final decision to include any paper that met the inclusion criteria in the review was made by group consensus. The doctoral researcher organised titles and abstracts for screening in Word documents and did not use any screening software to avoid introducing new programmes to the public advisors.

At the data extraction stage, the doctoral researcher extracted information from the included papers using the form that he initially piloted with public advisors. Subsequently, public advisors conducted their own data extractions, and these were then compared to identify any disagreements during the meeting. After data extraction, the doctoral researcher gathered the results and organised a meeting with public advisors. During this meeting, the doctoral researcher shared some results, and after each section, public advisors discussed their relevance to the review aim and their implications for both researchers and the members of the public. Again, public advisors offered a public perspective. For example, they compared the logic model on public involvement in big data research that was developed as a part of the review with their personal experiences as public advisors. They reflected that the initial model did not recognise that public advisors could face involvement barriers (such as complex language) when being involved in big data research; this was addressed with the revised model.

### 2.2 Interviews With Polish and South Asian Communities

**Figure 1:**
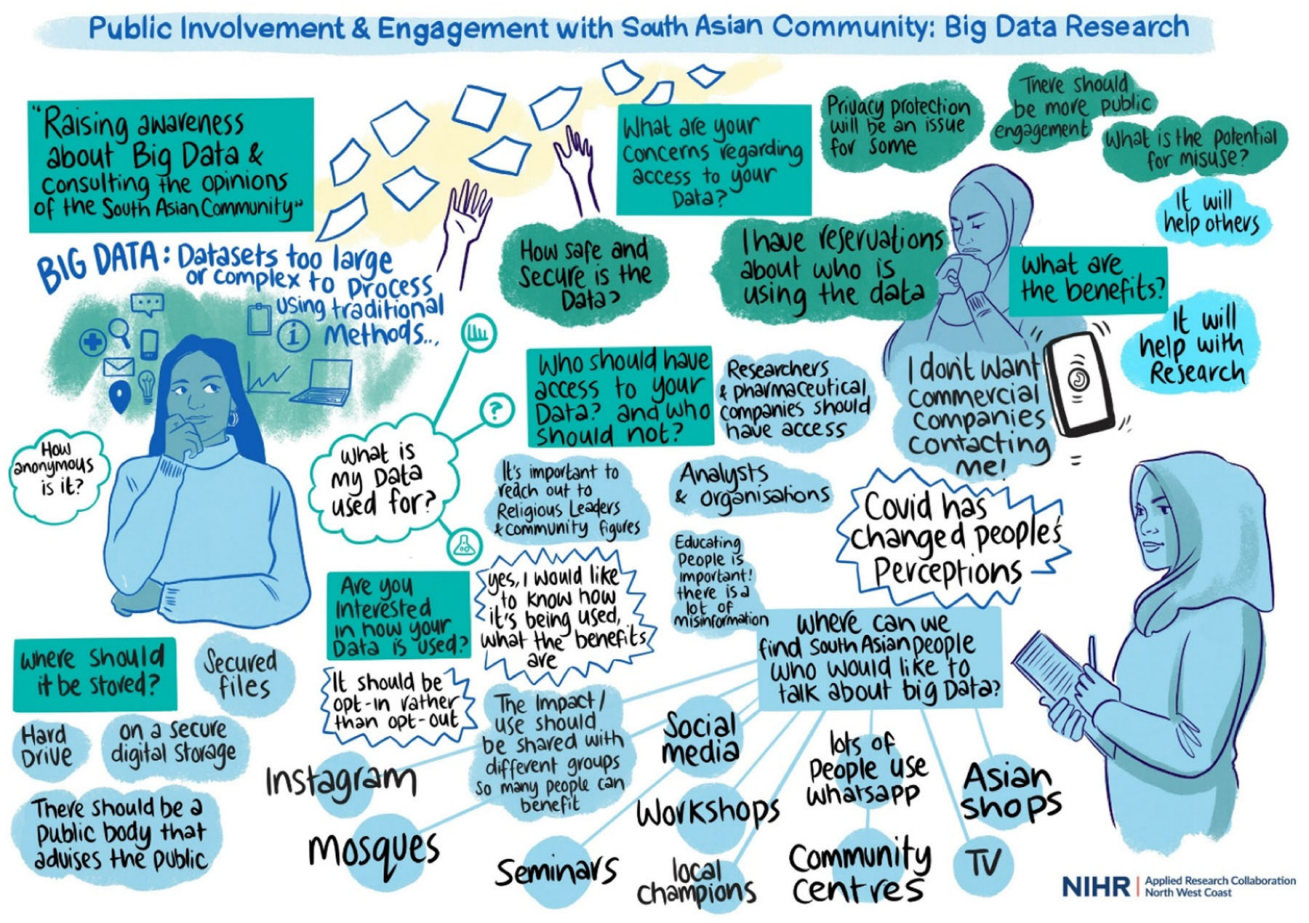
Visual minutes from an additional public involvement session with the South Asian community. Illustration by Ada Jusic.

**Figure 2:**
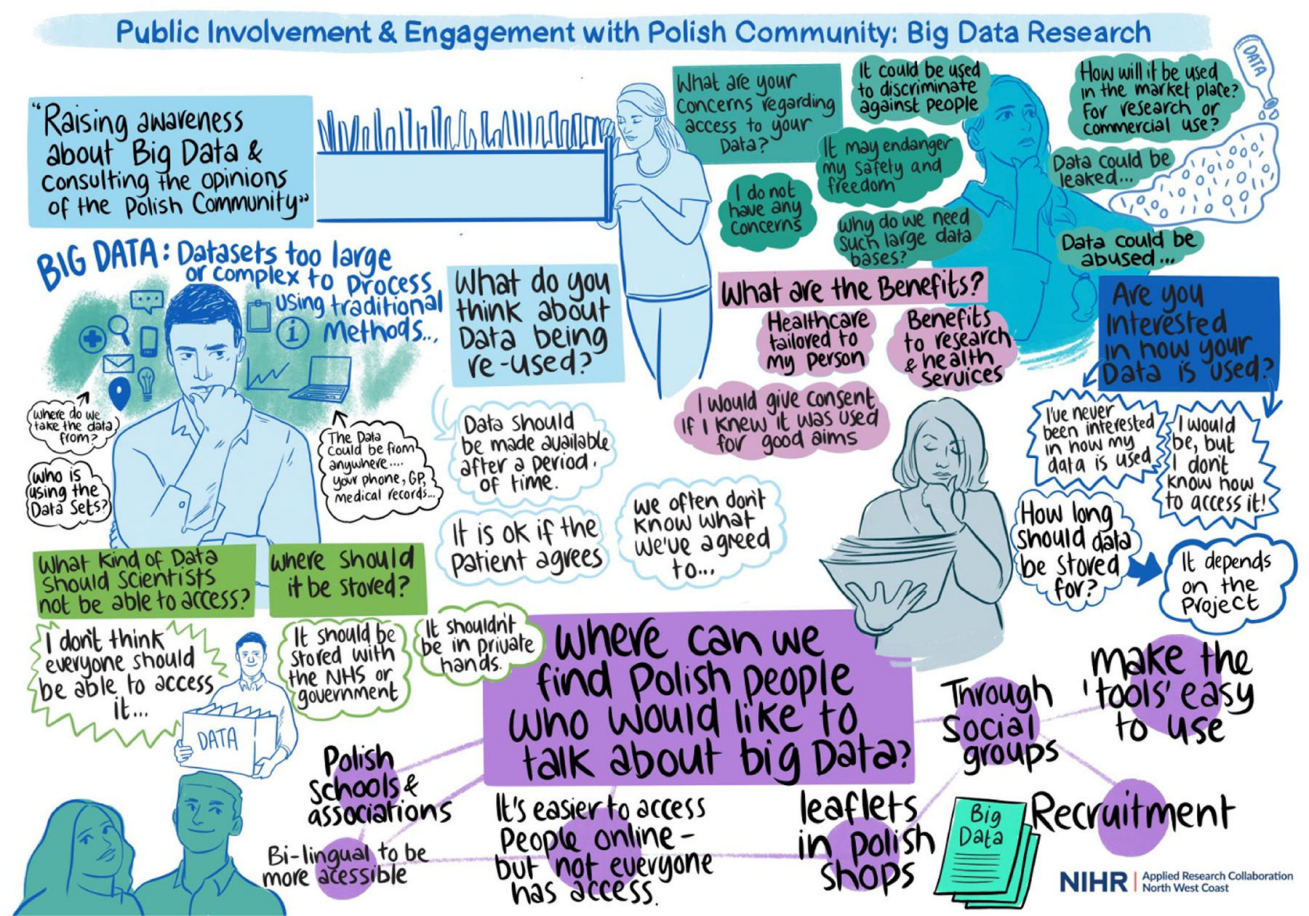
Visual minutes from an additional public involvement session with the Polish community. Illustration by Ada Jusic.

Public advisors were involved from the design stage of this qualitative research that aimed to understand how Polish and South Asian communities could be involved as public advisors in big data research projects [17]. Usually, joint meetings between the doctoral researcher and both public advisors were held. However, due to time commitments, it was not always possible to meet both public advisors each time [5]. When that happened, the doctoral researcher held one‐on‐one meetings. As qualitative studies require ethical approval, public advisors were involved in the ethics application. They reviewed interview guides and consent forms, contributed to the study design and supported the recruitment for broader public involvement. The ethics application was submitted at the end of the first year, so public advisors felt that they had become familiar with big data research. This made them wonder if everything in the topic guide was written in an accessible language or if they easily understood it because of their familiarity with the research. Thus, the decision was made to run another two group discussions with the members of the public not involved in the thesis to pilot the interview questions. Public advisors were involved in co‐designing them. The South Asian group was co‐chaired with one of the public advisors. However, as the Polish group was conducted in the participants' mother tongue (which the public advisors do not speak), the doctoral researcher asked for help from a Polish speaker experienced in public involvement from one of the charities working with migrants and ethnic minorities in the United Kingdom. These two public involvement sessions took place online but were designed to be engaging and interactive, for example, through visual minutes, padlet and polls [26]. Figures 1 and 2 show the visual minutes summarising the discussion. The visual minutes were developed live during the sessions by a professional illustrator and were approved by members of the public attending. These sessions ensured that all interview questions were in an accessible language; thus, later, interviewees felt comfortable answering these questions.

Once again, the doctoral researcher asked public advisors if they would like to be involved in other stages of the study. Public advisors requested an estimation from the doctoral researcher regarding the duration of various research activities. Based on this estimate, they decided the extent of their involvement. Consequently, they participated in coding one interview for each participant group (Polish and South Asian) and in defining themes.

Evidence in the literature shows that public advisors can contribute to qualitative analysis [27]. For example, Hemming et al. [24] reflected on the process of training and involving one public advisor in thematic analysis (following Braun and Clarke's approach [28] that was used in this thesis). One thing that they noticed was a significant overlap between codes identified by public advisors and the researcher. Similarly, codes generated by this doctoral researcher and public advisors were closely aligned.

In line with the established process, the doctoral researcher delivered training on reflexive thematic analysis [28, 29]. During the session, he explained the coding process. Then, the doctoral researcher and public advisors jointly coded an extract from one of the interviews and reflected on what and why they coded these sections in reference to the research questions. Afterwards, they separately coded one interview and came back together to discuss codes. This was also used to reflect on whether the interview guide was appropriate. Rubin and Rubin [30] recognise that asking every follow‐up question is not always possible as an interview has time constraints. The interviewer has to decide which avenues of inquiry should be followed and choose those most relevant to the research questions. Public advisors assisted in the process of identifying key issues for exploration with participants. In the coded interview, the interviewee suggested that religious events could be a good place for researchers to engage communities in big data research. Public advisors questioned the appropriateness of this method and suggested exploring this issue with other participants. Thus, the doctoral researcher asked the following interviewees who identified themselves as Muslims about their views on it. This approach allowed for the elicitation of the complexity of engaging during religious festivities.

When the doctoral researcher completed coding the remaining interviews and organised the initial themes, these were sent to the public advisors and supervisors. Through an email exchange, it was decided to have a joint meeting bringing public advisors and supervisors together to discuss the initial results. Based on that discussion, the doctoral researcher drafted a conference abstract [31] and a paper [17], both of which public advisors co‐authored. The conference abstract was accepted, and additional funding was received for one public advisor to co‐present the results with the doctoral researcher at the international conference.

### 2.3 Interviews With Researchers

The qualitative study, which explored the researchers' perspective on how to involve and engage seldom‐heard communities in big data research [16], was conducted alongside interviews with Polish and South Asian participants. Consequently, public advisors were involved in its design and in the ethics application as it was a joint one. Following a similar process for the analysis, public advisors coded one interview. Again, they identified additional issues for exploration. One participant spoke about the seldom‐heard community, but it was unclear how they would define their public advisors as seldom‐heard. This was explored as an additional follow‐up question during other interviews. As the analysis took place during the summer period, arranging a joint meeting between supervisors and public advisors was challenging, so the initial themes were discussed in separate groups. Thereafter, the doctoral researcher developed the themes and drafted the paper, which public advisors co‐authored.

### 2.4 Positionality of Public Advisors

In qualitative research, researchers' positionality could influence how they perceive and interpret the data; thus, there have been calls for researchers to recognise their biases and be reflexive [28]. Public advisors, too, can be biased in their opinions [32]. Limited literature discusses how public advisors' experiences influence what they perceive in data. However, some concerns exist that strong‐minded individuals could attempt to bring their agenda to research [27, 33] This is especially recognised in activist research, where multiple identities can exist (e.g., being a researcher and an activist) [34]. Public advisors share their lived experiences and are influenced by them (similarly to researchers). This potential bias could impact the quality of research. To overcome this challenge, the doctoral researcher provided training around reflexivity for public advisors. It focused on the importance of reflection and how one's experiences, backgrounds and personal views could affect what one perceives in data. During that training, the doctoral researcher shared his positionality, which was followed by public advisors reflecting on their own. Afterwards, public advisors asked for some questions to consider when analysing interviews. These were influenced by the work of Helen Kara [35]:

1. What views, perspectives and opinions do I bring to the discussion?
2. Are my views similar or different to the participants' experiences?
3. Are there other perspectives to understand what participants said?

Each analysis meeting started with a short conversation about public advisors' initial thoughts on the data and their positionality towards it. This approach was similar to Hemming et al. [24], who provided the public advisors with a reflective guide consisting of 20 questions as guidance. The impact of our training was visible during meetings. Public advisors started to question why they looked at the data extract in a particular way and considered their reactions towards it. For example, in relation to the previously mentioned example of religious events, they linked their personal experiences and considered the participants' answers. As the public advisors came from South Asian communities, they offered an insider view into this participant group. This was a unique perspective, as neither the supervisory team nor the doctoral researcher could have provided those insights.

## 3 Evaluation

The evaluation took place during an online meeting attended by the doctoral researcher and two public advisors. Having worked closely for the past 3 years, they agreed to evaluate the involvement process jointly. Adhering to UK Standards for Public Involvement [36], in this evaluation, the doctoral researcher and two public advisors were equally involved in the analysis and write‐up.

Public involvement could be seen as a complex process. Therefore, the established public involvement evaluation framework by Gibson, Welsman, and Britten [11] and Gibson, Britten, and Lynch [12] guided the process of mapping experiences and evaluating public involvement in doctoral research. The framework is four‐dimensional. First, the framework recognises that not all voices in research projects might be equal. Thus, it identifies strong and weak public voices. The former refers to situations when public advisors provide feedback that influences research, whereas the latter means that their voice is of little consequence. Second, public advisors could be involved in one or multiple ways. Using only one approach might disproportionally strengthen one voice over another. The third aspect explores whether the discussion concerns the public or the organisation. In doctoral research, the organisation's concerns would include the university, the funder and the doctoral researcher. Lastly, the framework explores how easily the public advisors' feedback was implemented by the organisation.

Both public advisors were involved in other research (including doctoral), and one of them, SA, had prior experience using this framework. This method allowed for the recording of involvement interactions and for reflecting on differences in everyone's experiences. The results are presented in a diagrammatic and narrative way.

This work received ethical approval from the University of Liverpool Ethics Committee under number 12882. In accordance with established public involvement policy, public advisors were reimbursed for their time.

## 4 Results

**Figure 3:**
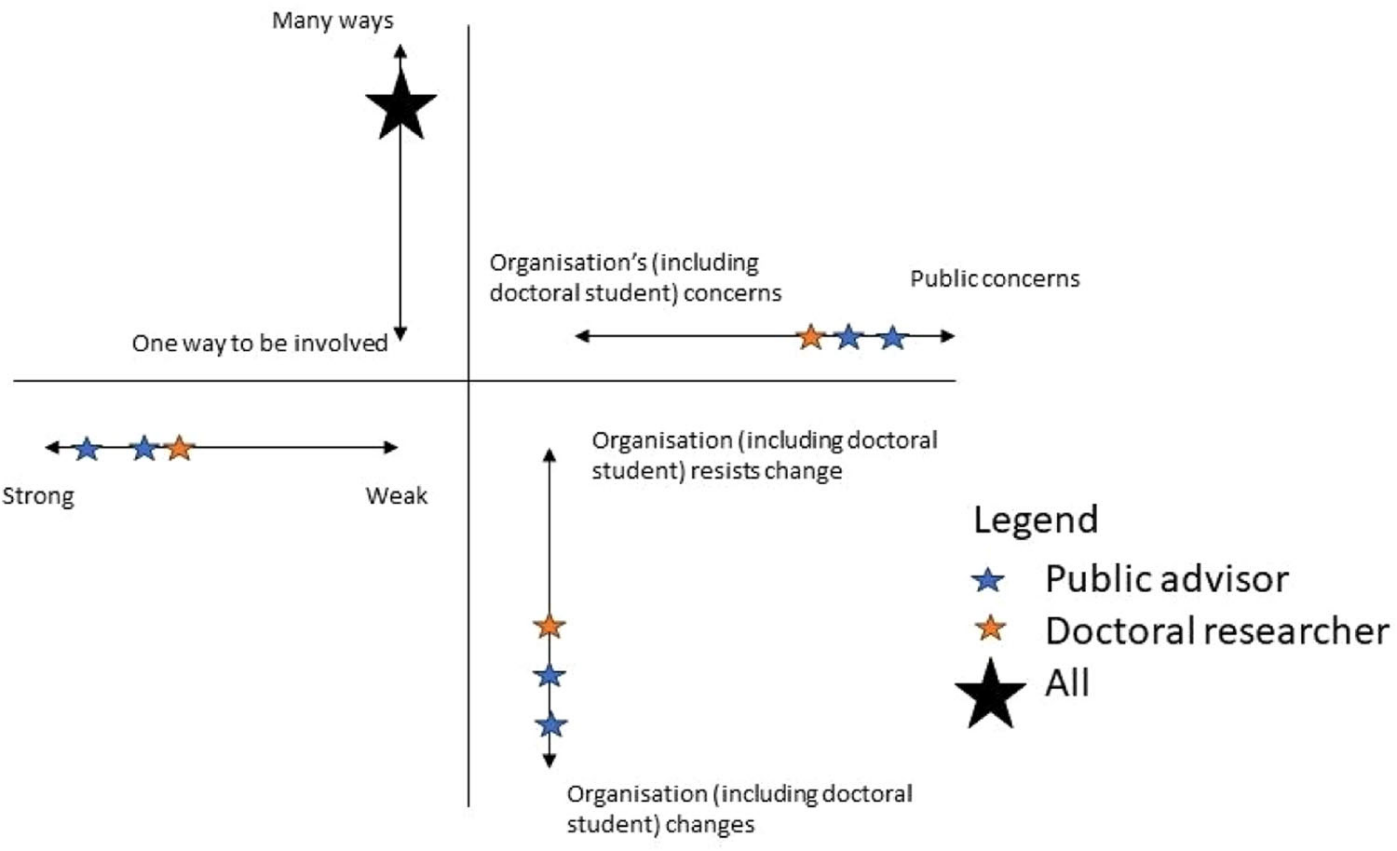
Results from the evaluation based on a four‐dimensional framework by Gibson and colleagues.

During the evaluation session, the doctoral researcher and public advisors individually mapped their experiences on the framework. Initially, these were mapped on four separate charts, each focussing on one aspect. Thereafter, they were put together in a diagram (see Figure 3). Now, the discussion is presented on what influenced the doctoral researchers' and public advisors' decisions to locate their experiences in each aspect of the framework.

### 4.1 Strong/Weak Voice

Responses leaned towards public advisors having a strong voice in doctoral research. N.T. located her experiences closest to a strong voice, feeling that she was an equal partner in the doctoral research. Similarly, S.A. also mapped her experience close to a strong voice but recognised the limitations imposed by doctoral research. S.A. argued that as a public advisor, she had to be realistic about the extent of her influence on shaping doctoral research, considering other voices, such as those of supervisors, which might carry more weight. The doctoral researcher agreed with S.A., and seeing that the topic of his doctoral work was chosen beforehand, he had limited scope to ensure the public voice shaping the thesis' research questions. However, he stated that his aim was to ensure that public advisors were co‐researchers as much as they wished to be involved, and it would be feasible within the constraints of doctoral research.

### 4.2 Many Ways/One Way to Be Involved

There was an agreement that public advisors had multiple ways to be involved in doctoral research. These methods included online and in‐person meetings, providing feedback outside meetings, such as on draft outputs, via email and engaging with each other on social media (Twitter). S.A. pointed out that the doctoral researcher provided training that facilitated the public advisors' ability and confidence to be involved in research. However, N.T. reflected that these were one‐sided as the doctoral researcher delivered (and designed) without the public advisors' input. For example, when the doctoral researcher delivered training on conducting thematic analysis, he initially consulted with public advisors to determine their familiarity with the approach. Based on their self‐identified needs, he prepared training materials and practical exercises. The materials and exercises were chosen based on available literature rather than further public advisors' input. Due to budget constraints, it was impossible to involve another public advisor experienced in that process to co‐deliver the training. Thus, the training consisted solely of the researcher's perspective.

### 4.3 Organisation's/Public Concerns

Everyone's experiences were mapped close to public concerns, thus recognising that the research focussed more on the public rather than the organisation's needs. S.A. placed her experience closest to public concerns but reflected that this was not always the case. When she initially joined the doctoral project as the public advisor, she perceived the research topic to be of more relevance to the organisation's concerns. However, as the projects were shaped and research was undertaken, she found that these became more and more relevant to public concerns. This view was shared in feedback from her fellow public advisors when the doctoral researcher presented some work during the Public Adviser Forum that this research was not only about what the researcher was interested in but also about public advisors.

The doctoral researcher reflected that the PhD thesis was supposed to be an independent and sole authorship piece of work. This restricted him from co‐writing the thesis with others. Thus, public advisors were included only in the acknowledgements section of the thesis, but they became co‐authors of academic papers. The initial scope of research was based on the description written by the supervisors and approved by the funder. However, the doctoral researcher found that the approach was not compatible with conducting research ‘with’ rather than ‘for’ the public. Consequently, he endeavoured to implement public advisors' views whenever possible.

### 4.4 Organisation Resists Change/Organisation Changes

There was an agreement that the doctoral researcher would implement public advisors' feedback into the projects, including the design, data collection and analysis. However, there were limitations to what was possible due to the structure of the doctoral programme. Both public advisors felt that their feedback (and sometimes criticism) was taken on board and led to real (and not tokenistic) changes. N.T. provided an example where she had asked to change the title of the paper, as she found it inappropriate. She saw it as a major change, which the doctoral researcher implemented. S.A. added that the change was not only about the research process but also the way the doctoral researcher worked with public advisors. For example, online meetings were initially supposed to be held on Microsoft Teams, but S.A. asked if these could be moved to Zoom as she was more familiar with it.

The doctoral researcher mapped his experience closer to the organisation's changes rather than resisting change but not as closely to public advisors. Again, the doctoral researcher reflected that his research was within the constraints of what a PhD ought to be. This was reflected in the joint meeting between supervisors and public advisors, where they discussed the initial results from one of the qualitative projects. Public advisors felt that their feedback during that meeting was heard but also recognised that implementing everything was not always possible. When this occurred, the doctoral researcher openly disagreed but also explained why this was the case and attempted to identify solutions together with the public advisors. Strong communication between the doctoral researcher and public advisors facilitated these changes and helped establish relationships over 3 years of collaboration, allowing them to be open with each other.

## 5 Discussion

Through the use of the four‐dimensional public involvement evaluation framework by Gibson, Welsman, and Britten [11] and Gibson, Britten, and Lynch [12], the evaluation showed the evolving nature of public involvement in doctoral research, highlighting the importance of collaboration and flexibility between the doctoral researcher and public advisors. Overall, public advisors and the doctoral researcher mapped their experiences closely to each other and aligned in one aspect in the same place (many ways to be involved). However, public advisors consistently positioned themselves closer to the scale of what could be considered meaningful involvement. They mapped their involvement in doctoral research to be stronger, closer to public concerns and more impactful than the doctoral researcher perceived it to be. The doctoral researcher and public advisors agreed that involvement was conducted in many ways to facilitate the process. The findings highlighted several key issues regarding public involvement in doctoral research. The public advisors desired a strong voice in the research process; this was facilitated by training and support delivered by the doctoral researcher. However, their involvement in doctoral research was limited by PhD–related restrictions like defining research topics by supervisors and the funder or the doctoral thesis being an independent piece of work. Further individual reflections on involvement and personal development are presented in Table 2.

**Table 2:** Individual reflections on involvement and personal development.

N.T. reflections	My involvement in this research has been an immense learning curve. Representing my community, especially the seldom heard silent voices has made an impact on my personal development as a public adviser. The researcher involved me from the beginning. He took into account any training needs along the way that were required to better understand and contribute to my research role. I always felt my voice was heard and that I was making a difference be it big or small. The confidence to be able to do this and speak up was indeed due to the support, discussions and training received throughout the research to understand data, analysing, scoping, coding, reviewing papers and so on. A highlight for me, in particular, was presenting the research alongside P.T. at the International Population Data Linkage Conference 2022. The audience feedback was much appreciated but more so having the opportunity of standing proud with P.T. highlights equality and equity at its best. Adjustments when needed were made over the course of the research to give me the tools and skills to be able to not only implement and contribute what I had learnt but also disseminate this valuable research on an equal platform.
S.A. reflections	In November 2020, I got the opportunity to be a public adviser for P.T. study, exploring public involvement of seldom heard voices in big data research. In our first meeting, I requested a short presentation on systematic reviews where PT discussed his review too and this way, he started his doctoral research.
	During P.T.'s PhD he gave me training on literature search and then I helped him on the review where I read the papers and helped him to remove some of the papers which were not relevant. I also reviewed the research papers for him. I advertised public involvement and engagement with South Asian communities, big data event. I really enjoyed co‐presenting with P.T. at conferences. I got co‐authorship in publications, which I am proud of. The thing I enjoyed the most was evaluating our work by Gibson's theoretical framework. I was very proud when P.T. received the student of the year award, which he deserved.
	I enjoyed throughout P.T.'s research journey, where I felt valued, got a better understanding of health research, built links with the community, gained confidence, experienced being working in a team, made friends, had training in literature search, coding, good communication skills and computing skills. I learned so many skills working with P.T., which I will utilise for the rest of my life.
P.T. reflections	As my doctoral research explored public involvement and engagement, I decided it would be appropriate to ensure ongoing and meaningful public involvement throughout my doctoral journey. Throughout these 3 years, I had an opportunity to learn from N.T. and S.A. as they shared insights from their community with me and offered a unique perspective on findings that I would not have noticed myself. As the involvement process was ongoing rather than a one‐off opportunity, I felt it allowed us to build a strong working relationship. Doctoral research can sometimes be a lonely journey. However, in my case, I feel that public advisors provided me with this additional level of support, guidance and friendship that helped me through it.

Public advisors saw themselves as having a stronger voice than the doctoral researcher perceived. One way to explain this disparity might be that public advisors compared their level of influence to their previous experiences of involvement in other doctoral projects, while the doctoral researcher compared it to the examples that he was taught during the public involvement training sessions, which focused on public involvement in research rather than doctoral research. This suggests the need for public involvement in research training tailored for doctoral researchers.

The public advisors had prior experience in participating in research projects and academic publications, which equipped them with the confidence to voice their opinions and challenge the doctoral researcher [19]. Whenever feasible, decisions were reached through consensus, although achieving consensus was not always possible [32]. Appropriate training in research topics and methods was essential in ensuring that the process was meaningful to both the doctoral researcher and public advisors. The doctoral researcher developed the training materials and identified textbook readings, which were shared with the public advisors as needed. The learning process primarily focused on practical skills. Public advisors were instructed on performing tasks and then engaged in joint activities with the doctoral researcher, such as screening in reviews.

The main barrier was about asserting ownership over doctoral research in the context of public involvement [6]. PhD research is supposed to be a doctoral researcher's work, but simultaneously, involvement principles require sharing power over the research process. The doctoral researcher aimed to integrate the public advisors into the research team, fostering a sense of being co‐researchers and equal partners. This approach was intended to reflect the collaborative nature of the involvement process [37]. This was in contrast to some other involvement in doctoral research where public advisors acted only as a ‘patient supervisor’, a member of the supervisory team who guides the work [6].

Public involvement has become established among some researchers but sometimes remains misunderstood or used in tokenistic ways. Meaningful involvement requires researchers to believe that public involvement would benefit their research and improve the chance of publications and career progression. Writing a doctoral thesis differently than the established approach might lead to mixed opinions among peers [38]. For example, this might be the case when doctoral researchers actively involve public advisors throughout their research. If the researcher is in a supportive environment towards public involvement, there is a higher chance that public involvement will take place and be meaningful to researchers and public advisors. Boylan et al. [39] interviewed researchers to understand their experiences with public involvement. Some of their participants described their colleagues' views around public involvement as cynical, sceptical or ambivalent. Therefore, the organisational culture and support from senior researchers for public involvement can significantly influence how involvement is conducted and its impact [39]. Supportive supervisors can facilitate the public involvement process [32]. In this doctoral research, the doctoral researcher's peer network also included public advisors involved in their own research and supervisors who had experience working with the public; therefore, they were very supportive of public involvement.

Public advisors need to be recognised for their involvement and feel valued [19]. Following the National Institute for Health and Care Research's (NIHR) guidance [40], public advisors were reimbursed for their involvement. However, financial incentives are often not the main driving factors for those involved in health research [19]. During this doctoral research, the value of being involved was not only monetary, as public advisors had an opportunity to learn new skills, be co‐authors on papers and blogs and co‐present research results at conferences.

The doctoral researcher developed his own unique scholarly identity while working on the thesis [41]. However, public advisors ensured that the doctoral researcher reflected on the relevance of his research to the broader public. This is particularly evident in instances of co‐authoring or co‐presenting with public advisors. Peer writing with fellow doctoral researchers offers an opportunity to enhance academic skills, especially writing and critical thinking, as ideas are exchanged with similarly minded colleagues [42, 43]. Working with public advisors can have a similar impact on the doctoral researcher's development as a scholar and can foster the creation of new ideas.

### 5.1 Strengths and Limitations

The four‐dimensional framework enabled the doctoral researcher and public advisors to map their experiences systematically. However, some limitations should be addressed as the work was jointly self‐evaluated. First, it is possible that not everyone might have been comfortable sharing negative experiences with each other—as it was not possible to offer anonymity, as authors had previously published papers and presented at the conferences. However, at the beginning of the evaluation meeting, S.A. (as familiar with the framework) reminded the other public advisor and the doctoral researcher that they should remain critical and honest with each other. The established a working relationship over 3 years and the conclusion of doctoral research might have further facilitated open discussion. The second limitation of this paper is that it focused only on one research journey. Despite available literature on public involvement in doctoral research, none used the four‐dimensional framework, so comparing the results from the evaluation was impossible. Other doctoral researchers could use this framework when reflecting with their public advisors on how they were involved in their work. This could benefit doctoral researchers in the same departments, as the results identified structural challenges.

## 6 Conclusions

This paper has evaluated the involvement of two public advisors in doctoral research, which explored methods for involving the public in big data research. Their involvement ensured that the research was more relevant to the public. Public advisors influenced the study design (e.g., interview guide, consent forms) and the recruitment strategy and helped to provide a more nuanced analysis. The ongoing involvement also shaped the doctoral researcher's thinking and perspectives, as he learnt from the public advisors. Public advisors had an active role as co‐researchers or critical friends in this doctoral research. However, this was within the constraints of doctoral research.

## Author Contributions

**Piotr Teodorowski:** conceptualisation, formal analysis, writing–original draft, methodology, writing–review and editing. **Naheed Tahir:** conceptualisation, formal analysis, methodology, writing–review and editing. **Saiqa Ahmed:** conceptualisation, formal analysis, methodology, writing–review and editing.

## Ethics Statement

We received ethical approval to conduct this study from the Institute of Population Health Research Ethics Committee at the University of Liverpool (12882).

## Conflicts of Interest

P.T. is a member of the Health Expectations Early‐Career Researcher Editorial Board.

## Data Availability

Data sharing is not applicable to this article as no data sets were generated or analysed during the current study.
